# A Visit to the Dentist

**Published:** 1884-10

**Authors:** 


### A VISIT TO THE DENTIST.

BY ONE WHO HAS BEEN THERE.

"'Twas midnight's holy hour," and the great city, with all its burden of humanity, slept in peace. The pale moon rode the heavens in serene majesty. The stars looked down from their blazing thrones, and mocked the darkness. Great Orion was hunting with his dogs, and the seven Old Maid Sisters simpered at the unimpressible Hercules. Suddenly a cry tingled upward to the very vault of the sky, and re-echoed through all the arches of the empyrean—a cry which was like the agony of some strong soul in martyrdom—which voiced volumes of unutterable anguish—which spoke of rending heart-chords and snapping tendrils of the soul—a cry at which the shadows shuddered, the earth trembled, great Jove stared, and the rat-terrier howled piteously to silver-horned Diana.

Was it the crack of doom? Was it the day of dissolution? Was it the death-cry of one of the Immortals, which pierced through the shivering air, and shrieked through the silent streets, and awoke with its accents of woe the slumbering thousands from balmy slumber to acute sympathy for some appalling catastrophe? No! no insignificant matter like this, no such bare and pitiful circumstance sent such a pang through and through Mother Earth's bosom, and struck the babies like a cataclysm of green apples and cucumbers. It was something more portentous and tremendous in the awful and terrific consequences to the universe, and to humanity, and to Jones! For Jones had the toothache! Of all the somniferous millions peopling this degenerate planet, Mrs. Jones first became aware of the pitiful calamity as Jones bounded from the bed and came down on some freezing oil-cloth, clasping with both hands the cheek which enclosed the jumping, demoniac snag. And then all the other Joneses awoke. The baby was barely saved from an attack of catalepsy, and Bobby, who had been reading Buffalo Bill's thrilling romances, yelled in his sleep as he dreamed of the war-whoop of the Sioux, and felt their scalping-knives, and the whole household could not have been thrown into greater consternation, from centre to circumference, if some nihilist had blown them up with dynamite. And as for Jones, alas for all the weight of woe which the primal curse has brought upon the ruined race! Do not the problems of suffering, and the mystery of sorrow, and the crushing questions of sin and anguish which have baffled and oppressed the wisest for ages, become at times almost intolerable? There are crises in the history of every individual, when he is brought face to face with the great sphinx-like enigmas of life and its terrors, when life seems not worth the living, when earth is but a vale of tears, and any existence worse than none! Such a moment of destiny, upon which all his after years might turn, had come to Jones. He became suddenly and poignantly aware that living was not a jest; that our being must be something more than a terpsichorean feast; and as he danced around in the darkness, with the thermometer at zero, and barked his unprotected shins upon the furniture, Jones realized in all its profound solemnity that life was real, life was earnest! Jones was a pillar in the Hard-shell Baptist Church, and believed in foreordination, and predestination, and the perseverance and preservation of the saints, and his language on this memorable night was framed on the most orthodox models of the emphatic language of the strictest theology. It might not have sounded quite euphemistic and Chesterfieldian—it might even have had its Christian consistency questioned by some truly blue council of investigation—but there was no mistaking its significance. There was no doubtfulness about the utterances, such as characterize the words of the agnostic; there was a simple directness about it which avoided the weakness of all circumlocution, and expressed itself as pointedly and fervently as does eloquence in all ages. And Mrs. Jones, though horrified, was self-possessed enough to give her emphatically worse-half a piece of her mind. Jones was very glad it was only a piece, and not the whole, and trembled before the imagination of the remainder. But Jones himself was in no mood for jocularity. He felt the desperation of his circumstances, and yet he was conscious that, just as he was, he was hardly the figure for a heroic bust in marble. But Mrs. Jones was equal to the emergency. She always was. Jones had too often felt her guiding and shaping hand directing the current of his history, ever to doubt the competency of his wife. With some ejaculations peculiarly feminine she set to work in the cold to prepare a poultice, while her maniac husband shrieked at her slowness and cursed the man who first invented teeth. But Mrs. Jones was fearfully agitated by the gravity of the occasion, and by the precipitate cries of her husband, who felt that the emergency called for action, noble, sublime, godlike action, and in her haste the poultice was prepared so scalding hot that it blistered the cuticle of her good man by the time it had touched it. Ye who have seen the noble rage of the Spanish bull, pricked to exasperation by arrows and javelins; ye who have heard the mighty roaring of the caged Nemean lion as the old fat women have poked him with umbrellas in the circus, can but feebly imagine that howl of indignation, a sense of insult added to injury, as Jones executed a leg-movement which would have made a South Sea cannibal grow greenery-yallery with envy. There was a time in the halcyon days of lang-syne, in the coy and callow period of ecstatic youth, when Jones loved his wife, his heart's first choice. There was a time when he called her beautiful; a time when the appendix to the Great Unabridged, with its additional collection of endearing epithets, would have filled a long-felt want, as the giraffe said when he took a drink down through his mile of gullet. But so fades love's dream away; on this night his language could, on hardly any construction, be considered complimentary. It was more than merely negative; it was quite positive in its dialect of unappreciation. And Mrs. Jones thought that an extra bright diadem ought to be decreed to her for restraining her righteous anger under such a load of provocation. So she mildly apologized, and made a modest suggestion of toothache drops. A generous supply of the liquid was put into his mouth, but as it trickled and meandered down his esophagus it was a sight for gods and men. Jones acted like one of the ancient oracles who presided at the temple of Delphos, and his wife fled before his insanity only to be recalled in thundering tones with a demand for liniment. Perry Davis was speedily and tremblingly introduced, and without thought a dilution of that noted gentleman was applied to the already blistered surface of the face. Jones gasped at this next horror, for he was already exhausted. The climax was capped. He sat down upon the bed with smothered groans, like a man who had met his fate and mastered it. He would have come down to us, had he lived in those ancient times, as one of the grandest of the stoics. It is a consolation, in the direst extremity of our woe, to know at last that the worst possible has happened. We derive a comfort from the very absoluteness of our misery. Thus was it with Jones. He felt that he had touched bottom. He defied the gods to do their worst. He could smile at all their thunderbolts, like Prometheus chained to the rock and refusing to yield, though the vulture gnawed at his vitals.

At last Mrs. Jones ventured to say, timidly and laconically: "Why not have it out?" But this cutting of the Gordian knot did not especially commend itself to the sufferer. There are feelings in this world! Sentiment is not a thing to be made light of. The man who implored the woodman to spare that tree might not have been much of a poet; he may have been a namby-pamby individual, used to the melting mood, and dropping tears as fast as the Arabian tree its medicinal gum, but after all his heart was under the proper side of his vest. We dislike to part with old friends. It was a severe trial for that Congressman whose term had expired, when in a Washington boarding-house he was compelled to approach a venerable mackerel that had appeared so regularly upon the table, and take it gently by the tail for a farewell shake, as he tearfully exclaimed: "Good-bye, old mackerel! good-bye! The best of friends must part! This is a world of sorrow, where longest friendships must be ruthlessly severed! Good-bye, dear, old mackerel, good-bye!" And who, that has a spark of veneration in his bosom, can contemplate unmoved and unaffected the departure of some ancient molar that has seen our growth from youth to middle-life, an essential part of our existence; that has shared in the checkered scenes of our probation here below; that has by strict attention to the business of mastication filled its humble place duly and well, feeding stomach and brain, and keeping life and courage in the body; who can regard the taking away of such an integral and intimate part of us without a sigh of regret, tearful and deep? It is like losing a part of our identity. Something of self-consciousness seems to depart with it. We have a love for it which is a part of our ever-present self-love. And so Jones entertained some decided objections.

And is there not something in the loss of the teeth, one by one, which suggests too forcibly to be pleasant the final rack and ruin which shall befall the whole tenement of clay? The first visible intimations and potent arguments of our mortality, the first prophecy and proof of the oncoming dissolution is found there. Then first the shadow of death is cast over us, and we blanch to think that even so every bone, and cartilage, and muscle, and tissue shall decay, and the edict, "Dust to dust," go forth. Then first we shrink from the grim spectre with the scythe and cross-bones, as he sits on his throne of an extracted grinder and grins horribly as he cries: "Bye and bye I will make thy skull my seat." I cannot vouch that Jones went over all these sentimental considerations, but he was certainly solemn enough to have been cogitating on death and the final judgment.

By the time morning came, Jones emerged from the house pale and haggard, like a man who had just seen his tailor, or whose wife has presented him with twins. But there was a look of grim resolution in his face, as though he could meet, if need were, the very inquisition itself, with all its screws and stakes. He had seen the glaring advertisements of a dentist whose prices for strictly first-class filling were somewhat less than John Chinaman's for shoveling dirt. He seated himself in the chair with the cold comfortableness of one who has his life insured and his will made. And there ensued such a chipping, and scraping, and boring, and drilling, and rasping of nerves, that poor Jones prayed that he might depart in peace. Great drops stood on his forehead, and he writhed like a poor victim of hydrophobia. He saw whole constellations by daylight, but there were no angels in those heavens. He would have howled like the dog that bays at the moon, but Jones was a Christian and he reflected that even an old pagan philosopher would have kept silence in grim endurance. And so the plugging, and hammering, and tapping, and filling went on, and Jones felt that he was but a pilgrim here, and sighed, "Vanity of vanities, saith the preacher, all is vanity." On the theory of skim-milk here and cream in heaven, the more misery here, the more happiness there, the more groans here, the more laughter there, Jones thought that he ought to deserve a reserved seat above, and have all the houris of the Mahomedan paradise. And as that dentist flourished files and borers above him, and stretched his cheeks and lips, and pried his jaws to an enormous extent, and punctured his gums and struck corruscations of phosphorescence through his brain, and occasionally, by way of an interesting variety, sent his prong into the nerve of the eye-tooth, Jones wondered why it had never occurred to the religious imagination to represent horned and hoofed Beelzebub with forceps and plugger! But at last the contortion ended and the extortion began, for so Jones regarded it. He thought that that operator had had enough diabolical enjoyment out of his acute misery to fully compensate him.

So Jones went home to the bosom of his family, a perfect representation of Patience come down from her monument, where she had been so long industriously smiling at grief. His wife jested him on his woe-begone aspect, but he was in no temper for the excitation of his risibilities.

Then he betook himself to balmy slumber, nature's sweet restorer, who is in the habit of knitting up the raveled sleeve of care. But balmy slumber didn't have any knitting-work on hand that night; not to any very great extent. For, scarcely had Jones retired with the sublime self-consciousness that there were few men in the whole universe that night who drew comforters over such a virtuous form as his, when twitch, thump, that nerve got away with forty strokes to the minute. There are only forty thousand adjectives of all descriptions in the English language, and few men have a corner on them all, but it would have required the marvelous descriptive powers of a Micawber to correctly delineate the scene. Was Bedlam loose? Was Pandemonium come up to earth? Jones was, on religious principles, opposed to dancing, with the exception of such stately and decorous steps as David executed before the Ark, but the way he pranced forward and backward, balanced, chassezed, corners to middle, gentlemen to partners, all hands 'round, in quadrille, schottische, polka and german, would have shocked his fellow deacons into dumfounded horror. Then he remembered what Plato had said, that there were times when, and persons to whom, it were better to die than to live, and he reached for his hip-pocket. But the caps snapped, and after several ineffectual attempts to scatter his brains he cooled down, as his wife, who had surveyed the scene with speculation in her eye as to No. 2 and another bridal trosseau, sarcastically remarked, "It's no use, Jones. I wouldn't try again. You haven't got any."

So she dosed him heroically with morphine, and he was soon snoring like the seven sleepers of Ephesus all put together. But with morning the purgatory was re-enacted, and Jones, weary with suffering, began to look around the eyes like those who tarry too long at the lemonade with a stick in it.

But this time he resolved to spare no expense, to stop at nothing short of half his kingdom, but to patronize a man who knew his business, and "knowing, dared maintain" a decent charge. So he stepped into Mr. Timothy Tendertooth's office, and thus addressed him:

"Is it possible for you, sir, to pull a tooth without lifting a man out of your chair to the seventh heaven of unutterable agony, and hold him suspended on the end of your key-wrench while you dangle him between the sky and the earth and mop the floor with his hair, or beat the carpet with his legs? Is it possible for you to relieve a piece of wretched human flesh and not leave him as if a cyclone or a mule had entertained him unawares? Is it possible for a man to go through your hands and not find himself in some future state of existence, the exact locality depending much upon his previous life?"

The dentist's smile was childlike and bland. And soon Jones was under the sweet influence of the nitrous-oxide, delicious intoxication. He dreamed of the glorious freedom of his bachelor days, and that mothers-in-law were a lost art. He dreamed that he was a boy again, stealing apples as of yore, and he forgot that he was a deacon as his features smilingly relaxed. He dreamed that there were no more gas bills, no more lightning-rod agents, nor book canvassers, nor life insurance solicitors. He dreamed of that coming Golden Age when there shall be no more political jobbery, when the aldermen shall be superintendents of Sunday schools and the mayors shall lead class meetings, and the pickings and the stealings shall belong only to the elect. He dreamed that he had realized on his corner lots, that his mining stock had gone up out of sight, that his minister had piously resolved to preach thereafter only an hour. He dreamed that he ascended to heaven, and that St. Peter let him in without any cross-questioning, and that the chief occupation of the angels there was to immerse those who had only been sprinkled on earth. Just as he was about to sit down upon a flowery bank and sing himself away to everlasting bliss, he awoke from his sweet delusion, returned from his elysium to find himself still chained to existence in this mundane sphere, still seated in the dental chair with a benediction radiating over him and a great ivory root triumphantly displayed before him. And the doctor soothingly assured him that a more beautiful and serviceable one could be felted into the place that once knew it but should know it no more forever.

And when he felt that all his agony was over, hope again sprang exultant in his breast; the interest of life with all its variety and vivacity returned like a flood-tide. He had begun to love not man nor woman either, but now his fellow-beings never seemed so noble and amiable to him. He felt that he could mingle again with the great tides of existence, and secretly vowed as a thank-offering a new seal-skin to Mrs. J.

Then he delivered himself of the following eloquent panegyric: "Respected and honored Sir! To you I owe life, liberty and the pursuit of happiness! To you shall I look as a friend and helper in one of the direst emergencies and extremities that ever befell my lot. My remuneration to you utterly fails to express the tons upon tons of gratitude—1200 lbs. full coal weight, net—which I feel for you. I shall remember you to my dying hour, and expect to see as good a man as you in Eden's rosy bowers. If I should ever get to Congress I shall have you appointed inspector of hydrants. It may not be that teeth shall ever ache in the future, but it is a principal part of my anticipation of the happiness beyond to see you and your gas-bag on the other shore.

"Sir! you belong to a noble fraternity! You take your place in the ranks of great philanthropists and humanitarians who have helped to save life or alleviate human misery! What were this life but a bitter struggle with the inevitable, what were our breath but vanity, what were all our labor but sorrow, were it not for men like you? An aching tooth, a shooting nerve, is a little thing like a thorn in the flesh, but it has power to afflict the soul and make our being a miserable thing.

"Sir! Go forward in a great career! May your shadow never grow less; may your fame never diminish; may your receipts never decrease. To-morrow I will send Mary Jones to have all her teeth out, and dream twenty-six times all the nice things that I have, and then while you are making a new set she can't talk for a month. And that will be heaven for me!

"Sir! Farewell! May the blessings of your contemporaries attend you. May you inspire new hope in posterity!

"Devote yourself entirely to your grand profession and you will not live in vain, and when you die the recollection of the hundred thousands of teeth you have pulled and filled will sweetly console you! Respected Sir! In the words of another, go on unto a great future! Be like an eagle and soar, and the sorer you get the more we shall be gratified!"

